# Formal Synthesis of (±)-Allocolchicine Via Gold-Catalysed Direct Arylation: Implication of Aryl Iodine(III) Oxidant in Catalyst Deactivation Pathways

**DOI:** 10.1007/s11244-017-0742-z

**Published:** 2017-04-19

**Authors:** Tom J. A. Corrie, Guy C. Lloyd-Jones

**Affiliations:** School of Chemistry, University of Edinburgh, Joseph Black Building, David Brewster Road, Edinburgh, EH9 3FJ UK

**Keywords:** Allocolchicine, Direct arylation, Gold, Catalysis, Deactivation

## Abstract

**Abstract:**

A concise formal synthesis of racemic allocolchicine has been developed, centred on three principal transformations: a retro-Brook alkylation reaction to generate an arylsilane, a gold-catalysed arylative cyclisation to generate the B-ring via biaryl linkage, and a palladium-catalysed carbonylation of an aryl chloride to generate an ester. ^1^H NMR monitoring of the key gold-catalysed cyclisation step reveals that a powerful catalyst deactivation process progressively attenuates the rate of catalyst turnover. The origins of the catalyst deactivation have been investigated, with an uncatalysed side-reaction, involving the substrate and the iodine(III) oxidant, identified as the source of a potent catalyst poison. The side reaction generates 1–4% of a diaryliodonium salt, and whilst this moiety is shown not to be an innate catalyst deactivator, when it is tethered to the arylsilane reactant, the inhibition becomes powerful. Kinetic modelling of processes run at two different catalyst concentrations allows extraction of the partitioning of the gold catalyst between the substrate and its diaryliodonium salt, with a rate of diaryliodonium salt generation consistent with that independently determined in the absence of catalyst. The high partition ratio between substrate and diaryliodonium salt (5/1) results in very efficient, and ultimately complete, diversion of the catalyst off-cycle.

**Graphical Abstract:**

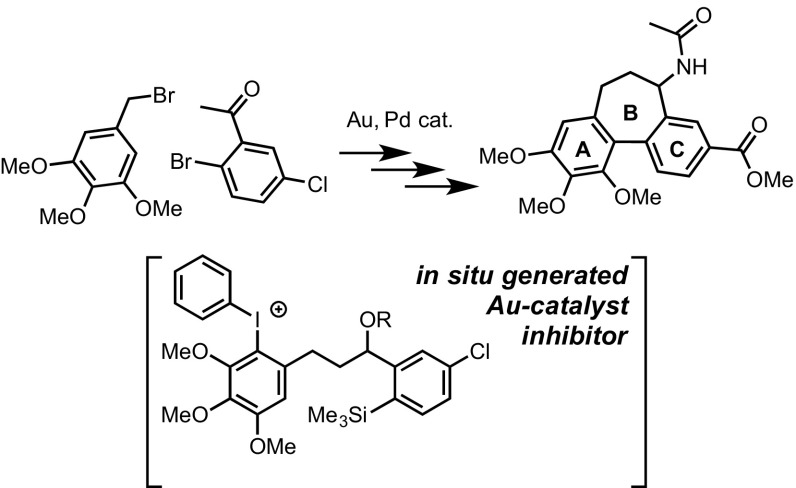

## Introduction

Novel catalytic C–C and C–X bond-forming reactions offer numerous opportunities for potential application in the pharmaceutical, agrochemical, and fine chemical industries. Such reactions are of particular utility when they are orthogonal to existing reactions, providing new avenues for chemical diversification. In the context of the development of new catalytic reactions, their application to relatively more-complex molecules can provide extremely valuable insight in addition to simply the challenge of the synthesis of the target. Indeed, they can highlight unforeseen problems with catalyst chemo-, regio- (and perhaps stereo-) selectivity, as well as other critical aspects relating to catalyst stability, efficiency, activity and functional group compatibility. Thus, beyond the simple substrates that are frequently employed for initial reaction discovery, complex molecule synthesis might be viewed as an essential component in testing for catalyst robustness.

**Scheme 1:**
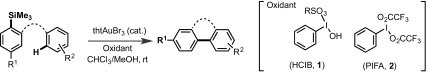
Reaction conditions for inter- and intramolecular direct arylation reactions. RSO_3_ = camphorsulfonate. HCIB is hydroxy(camphorsulfonyloxy)iodobenzene (**1**) and PIFA is ([bis(trifluoroacetoxy)iodo]benzene (**2**). A mixture of iodobenzene diacetate and camphorsulfonic acid can be used where HCIB is formed in-situ

In 2012 we reported a new C–C bond-forming reaction involving the coupling of arylsilanes with arenes, using a gold(III) catalyst in the presence of a stoichiometric ArIX_2_ oxidant, Scheme 1 [1]. The methodology was initially established as an intermolecular process, and the mechanism shown to involve sequential auration of the silane then arene, followed by reductive elimination from a diaryl gold intermediate [2]. Itami and Sagewa subsequently reported that Au-complexation by a pyridylidene ligand facilitates arylation of isoxazoles, indoles and benzothiophenes [3], and Jeon applied the gold-catalysed process to functionalize *ortho*-silyl aryl triflates generated via Rh/Ir-catalyzed traceless *ortho*-CH silation [4]. We have also expanded the process to include the intermolecular arylation of a broad range of heteroarenes at room temperature [5], and developed intramolecular arylations to generate 5–9 membered rings [6]. Of the more than 140 examples of the Au-arylation reaction studied to date [1–6], the majority proceed efficiently with low catalyst loadings (typically 1–2 mol% Au) under mild conditions, and quite frequently at ambient (20–25 °C) temperature.

**Scheme 2:**
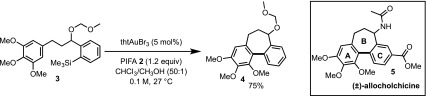
Cyclisation to generate an allocolchinoid skeleton (**4**)

The intramolecular process [6] allows the use of a much larger scope of arene substrates, and facilitates the study of mechanistic details [2] that cannot be elucidated in the intermolecular reaction. In the case of electron-rich arene substrates, replacing the “HCIB” oxidant (**1**), with “PIFA” (**2**) substantially reduced the rate of competing diaryliodonium salt formation [7], leading to greater yields of the desired C–C coupled cyclisation product. In the case of the cyclization of **3** to **4**, where both the substrate and product bear a highly electron-rich trimethoxy benzene ring (Scheme 2), use of PIFA was essential.

Herein, we present further advancement of this gold-catalysed ring-forming process by way of a formal synthesis of (±)-allocolchicine (±)-**5** [8–11], utilising the orthogonality of the process to palladium catalysis to install the requisite ester via carbonylation [12]. The synthesis has revealed a potent mechanism of catalyst deactivation, with substantial implications from this for the requirement of a change in the nature of the oxidant in further developments of the methodology.

## (±)-Allocolchicine

The gold-catalysed synthesis of (±)-allocolchicine begins with commercially available ketone **6** 1. After conversion to a silyl enol ether, **7** [13], this is engaged in a retro-Brook rearrangement [14] and the resulting lithium enolate alkylated by trimethoxy benzylbromide **8** to afford *ortho*-silyl arylketone **9** [15]. Reduction to alcohol **10**, then *O*-alkylation with MOMBr leads to the pre-cyclisation scaffold **11**. Under the conditions employed for **3** (Scheme 2) this undergoes gold-catalysed cyclisation to give **12** in 56% yield (with 24% recovered starting material **11**). Palladium-catalysed carbonylation of **12** gives **13** in 70% yield. Ester **13** connects with the synthetic pathway of Fagnou and Leblanc [9]^2^, thus completing a 10-step formal synthesis of (±)-allocolchicine (±)-**5**.

**Scheme 3:**
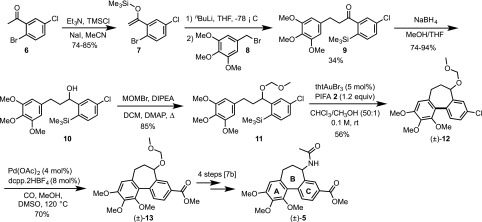
Formal synthesis of allocolchicine, (±)-**5**. dcpp = 1,3-Bis(dicyclohexylphosphino)propane

Our initial strategy involved cyclisation of ketone **9** or alcohol **10**, to give **14** or **15**, which on carbonylation (Scheme 3) would give ketone **16** or alcohol **17**, both of which are on the synthetic route developed by Wulff et al. [8]. This would provide a more concise and protecting-group free strategy. However, in contrast to MOM-protected **11**, neither the ketone **9** nor alcohol **10** underwent gold-catalysed cyclisation.

**Scheme 4:**
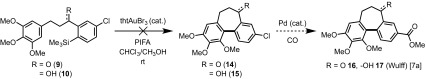
Attempted formal synthesis using ketone **9** or alcohol 10 to access compounds **17** and **18** on the synthetic route developed by Wulff [8]

**Scheme 5:**
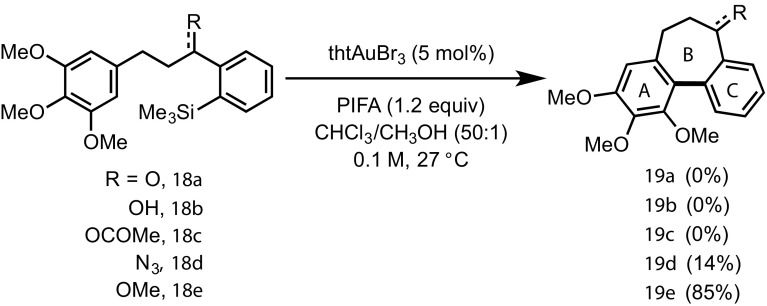
Reactivity of **18a**–**e** in gold-catalysed direct arylation reaction. Yield determined by ^1^H NMR [16]

To further explore the impact of the ‘R’ group (Scheme 4) on the cyclisation process, several derivatives of the non-chlorinated analogue **3** were prepared (Scheme 5). Only azide **18d** and methyl ether **18e** underwent successful cyclisation. It is clear from these observations that the identity of the ‘R’ group, and possibly its propensity to coordinate to gold, has a major impact on the success of the reaction.

## Catalyst Deactivation

**Fig. 1:**
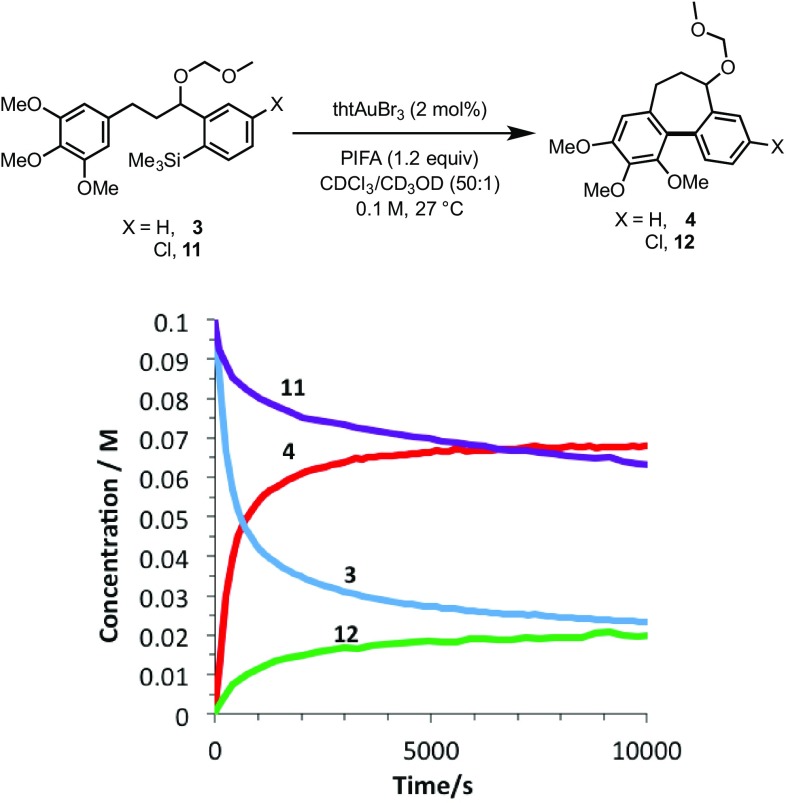
Comparison of temporal concentration profiles (in situ ^1^H NMR analysis, see SI for full details) for the gold-catalysed oxidative cyclisation of **3** and **11**

**Fig. 2:**
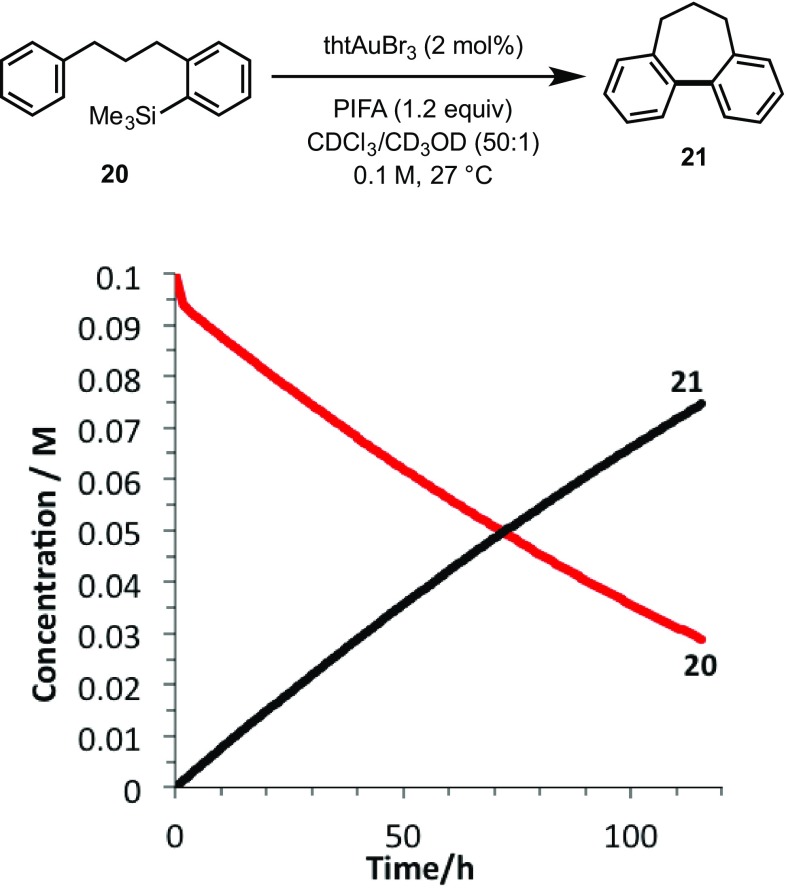
Temporal concentration profile for the cyclisation of **20**–**21**. The initial (non-productive) burst of consumption of **20** arises from the catalyst activation process in which two gold-derived bromonium ions are trapped by the substrate [2]

Although the formal synthesis of (±)-allocolchicine (**5**) was achieved, the 56% yield of **12** obtained from the cyclisation of **11** is low, especially considering that 5 mol% Au is employed. To gain a better understanding of the implications of using these more complex substrates in the direct arylation reaction, the kinetics of cyclisation of **3** and **11** using 2 mol% Au were monitored by ^1^H NMR (Figs. 1, 2). The temporal concentration profiles are indicative of severe catalyst deactivation: after initial rapid turnover, both reactions stall, with significant amounts of starting material remaining. Notably, the reaction of chloroarene **11** stalls significantly earlier than that of **3**, after only *ca*.20% conversion to **12**.

In order to assess whether the catalyst deactivation is innate to the use of PIFA, the kinetics of cyclisation of the ‘defunctionalised’ substrate **20** were monitored under the same conditions. Even though the cyclisation of **20** is very slow compared to the initial rate of turnover using **3** and **11**, the kinetic profile, which is approximately pseudo-zero order [6], indicates that there is no significant progressive catalyst deactivation, even after 100 h, as compared to **3** and **11** where catalyst deactivation is extensive within 2 h.

**Scheme 6:**
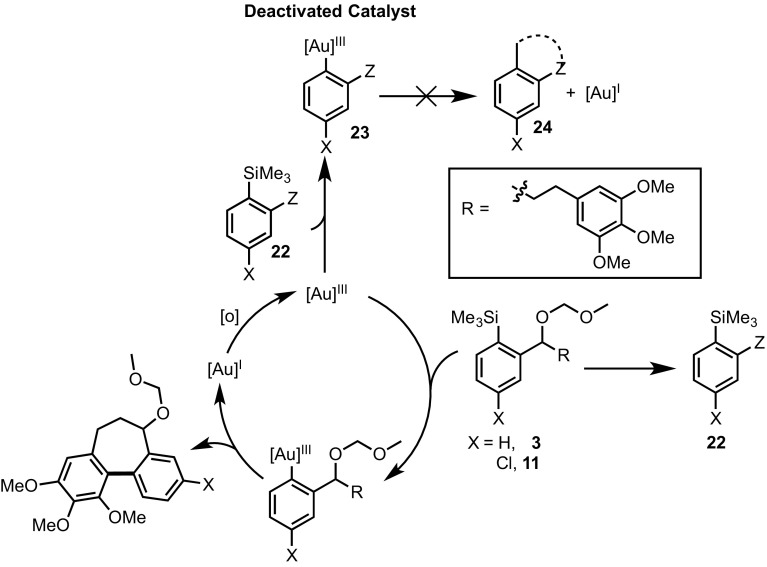
Generic catalyst deactivation mechanism involving in situ generation of competitive inhibitor **22** via in situ modification of side-chain functionality (CH(MOM)R → Z)

The difference in substrate structure between **3**/**11** and **20**, is the absence of both the MOM ether and a highly electron-rich arene in **20**, suggesting that the cause of deactivation is due to the presence of one or both of these functional groups. We thus considered a variety of generic catalyst deactivation mechanisms, including that shown in Scheme 6. Here, a side reaction, involving the side-chain (‘Z’) functionality, converts substrate (**3** or **11**) into an inhibitor (**22**), which then undergoes competitive transmetalation with the gold to generate an off-cycle complex **23**. If this species is unable to cyclise to **24**, or to reductively eliminate the biaryl product, and thus unable to release gold back on-cycle, then progressive catalyst inhibition will occur. The impact of the inhibition process will depend on the relative rate of reaction of substrate (**3**/**11**) versus the inhibitor (**22**) with the Au(III).

Our attention initially focussed on the MOM ether in **3** and **11**, as this protecting group functionality is, by design, acid-labile, and the reaction medium contains, or generates, CF_3_CO_2_H from the PIFA (**2**). Partial in situ cleavage of the MOM group (Scheme 6), to liberate alcohols **10** and **18b**, might then be a route to inhibitor species.

**Fig. 3:**
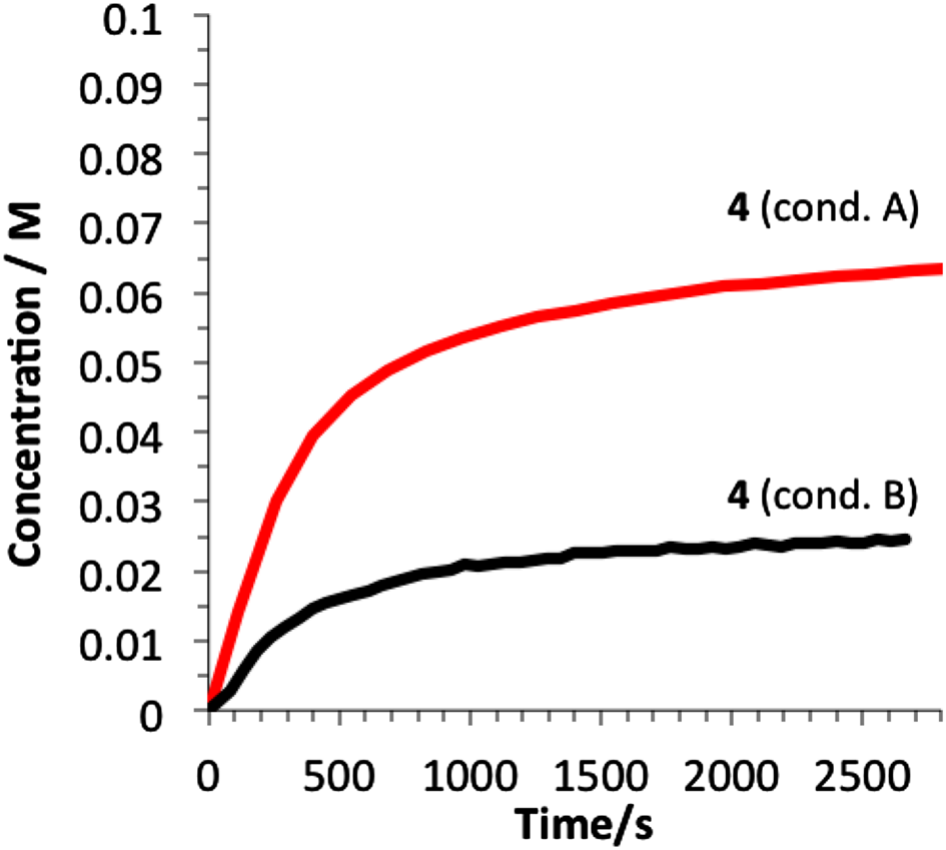
Conditions A: cyclisation of **3**–**4** under standard conditions (as Fig. 1) ; Conditions B: same a conditions A, but with 10 mol% **18b** added at the start of the reaction

As noted above neither **10** nor **18b** undergo cyclisation (see Schemes 4, 5). Moreover, inclusion of a catalytic amount of alcohol **18b** in the reaction of **3** resulted in an even earlier onset of catalyst inhibition (Fig. 3).

**Fig. 4:**
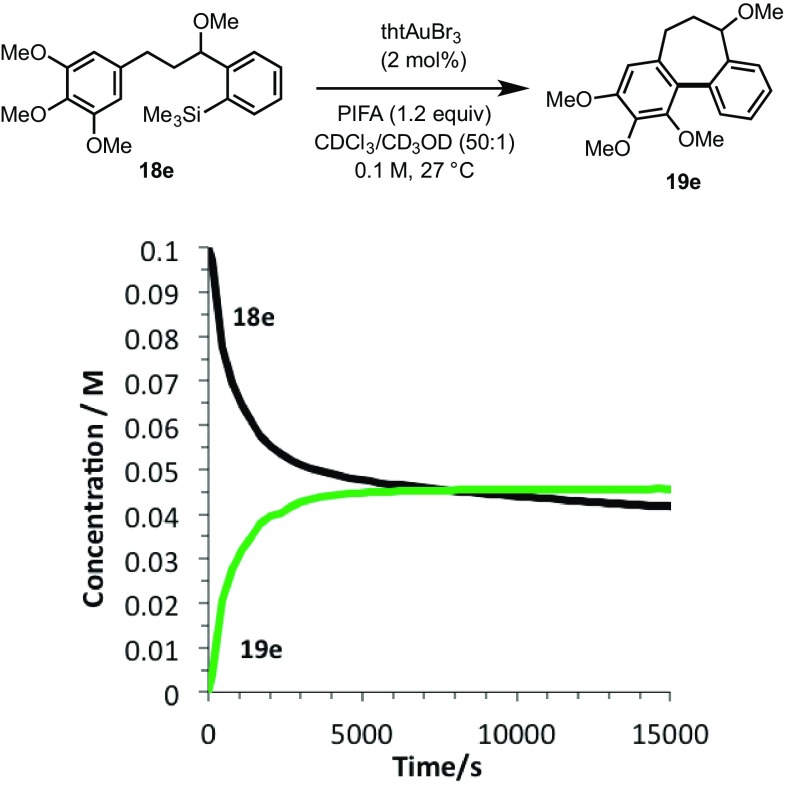
Temporal concentration profile (in situ ^1^H NMR analysis, see SI for full details) for cyclisation of acid-stable methyl ether **18e**, showing potent catalyst deactivation still occurs *without* the need for an acid-labile MOM protecting group (as in **3** and **11**, Scheme 7)

Whilst this confirms that alcohol **18b** can act as a catalyst poison, possibly by competition with **3** for the catalyst, and then strong off-cycle Au-chelation (**25**, Scheme 8), we were unable to isolate **18b** from the reaction, or detect any formation *in situ*. Consequently, the kinetics of cyclisation of the acid-stable methyl ether **18e** were monitored, with the expectation that no catalyst deactivation would occur if liberation of the hydroxyl group (**3** →**18a**) is required for inhibition. However, **18e** was found to undergo the same potent inhibition; indeed the initial rate and overall conversion (Fig. 4) was even lower than with the MOM ether substrate, **3**.

**Scheme 7:**
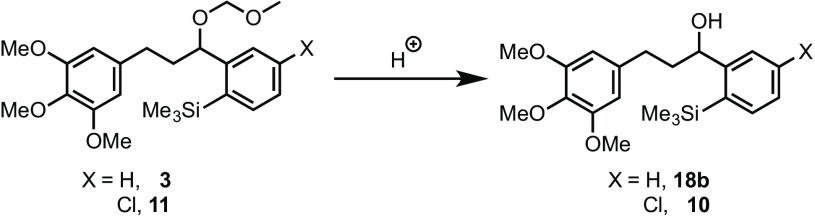
Possible in-situ deprotection of MOM protecting group under the reaction conditions

**Fig. 5:**
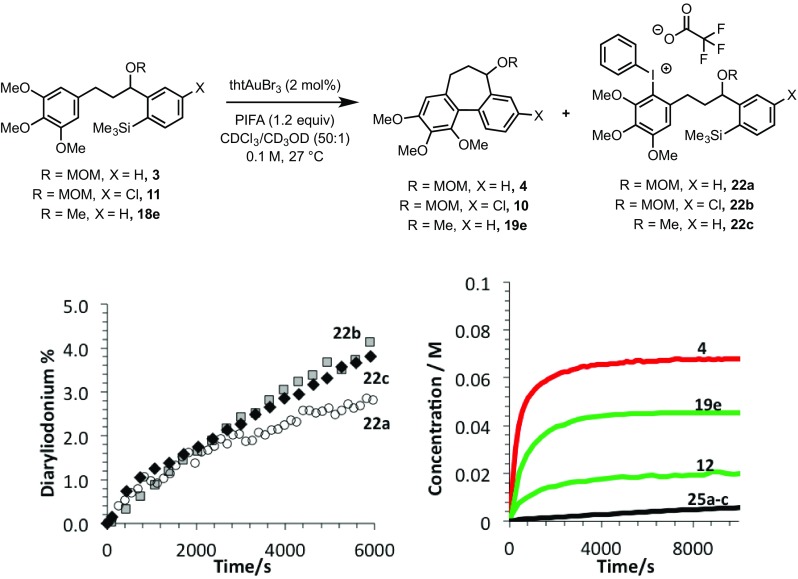
Formation of side diaryliodonium salt products **22a-c** under the conditions of cyclisation of **3, 11** and **18** to generate **4, 12** and **19e**

In further experiments, we confirmed that product inhibition of the catalyst was not the source of reaction stalling: addition of 100 mol% cyclised product **4** had with no detectable impact on the rate of catalyst turnover. Efforts were thus made to identify side-products in the reaction mixture of **3, 11** and **18e** that might behave as inhibitors. Whilst the reactions afforded satisfactory material balance, small quantities of side-products (**22a-c**) were detected by NMR. The rate of formation of **22a–c** was largely independent of the substrate (Fig. 5), and indeed catalyst loading. Highly electron-rich arenes are known to react with iodine(III) oxidants, such as PIFA, to form diaryliodonium salts, π-complexes or radical cations [17]. Careful in situ analysis of the reactions of **3, 11** and **18e** by ^1^H NMR indicated that **22a–c** are diaryliodonium salts; this was subsequently confirmed by mass spectrometry. The arylated trimethoxybenzene rings in the cyclisation products (**4, 12** and **19e**) are more hindered and less electron-rich than the non-arylated starting materials (**3, 11** and **18e**), and, within the limits of the in situ ^1^H NMR analysis, do not detectably get converted to the corresponding diaryliodonium species.

**Fig. 6:**
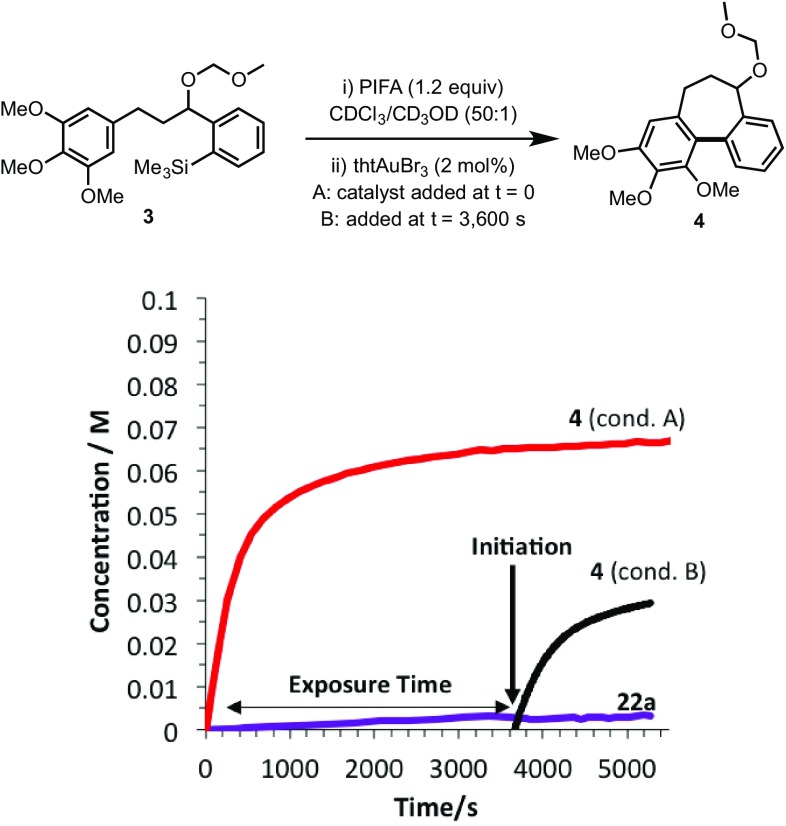
Conditions A: cyclisation of **3 to 4** under standard conditions (as Fig. 1); Conditions B: same a conditions A, but after pre-incubation of **3** with PIFA before initiating reaction

Evidence for the impact of substrate-derived diaryliodonium salt (**22**) generation was obtained by exposing substrate **3** to PIFA prior to addition of catalyst, allowing the build-up of the proposed inhibitor **22a**, and then initiating turnover by addition of the Au-precatalyst, Fig. 6. Significantly greater catalyst deactivation was observed in this case, indicative of a link between a reaction of the starting material with PIFA and the catalyst deactivation process^3^.

On the basis of steric hindrance and reduced electron density on the arene ring, diaryliodonium (**22**) generation would be expected to deactivate the trimethoxy-arene ring in aurated intermediates **23a**–**c** to aromatic electrophilic substitution, [7] and thus prevent either the cyclisation to **24a**–**c**, or reductive elimination of **24a**–**c**, and thus prevent release of gold (Scheme 9). In other words, it would not be the diaryliodonium salt generation *per se* that is poisoning the catalyst, but the result of tethering this salt to an arylsilane that can still undergo reaction with the gold catalyst (**22a**–**c** → **23a**–**c**).

**Scheme 8:**
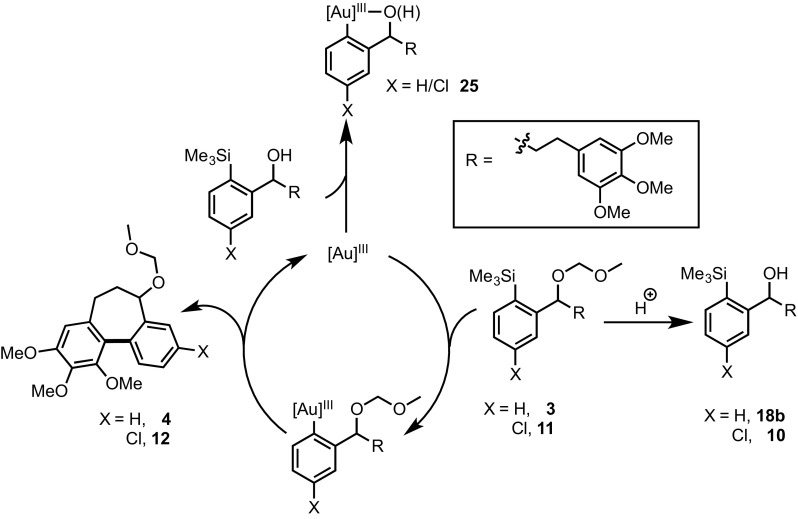
Possible mechanism for catalyst-deactivation by in situ generated alcohols **10** and **18b**

To further probe this aspect, trimethoxytoluene (**26**) was allowed to react with PIFA to form the diaryliodonium salt **27** (Scheme 10). Addition of 9 mol% **27** to the reaction of **3** resulted in no significant change in rate of turnover or inhibition, further reinforcing the concept that *tethering* of the diaryliodonium salt to the arylsilane is crucial in the proposed deactivation mechanism (Scheme 11).

**Scheme 9:**
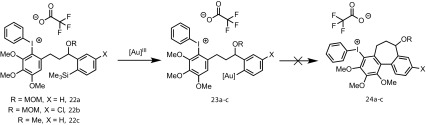
Tentative assignment of catalyst inhibitor, and associated deactivation pathway

**Scheme 10:**
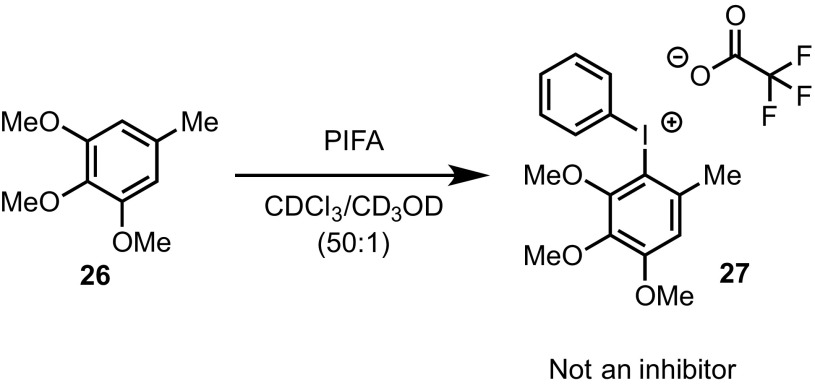
Preparation of diaryliodonium salt **27** for test as a catalyst inhibitor in the cyclisation of **3**–**4**

**Fig. 7:**
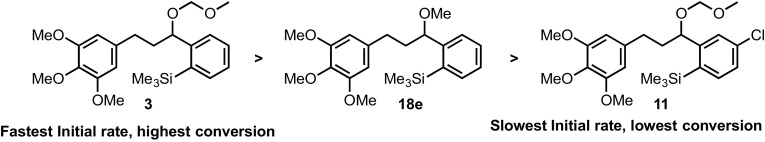
Substrate reactivity versus conversion

This deactivation mechanism is consistent with the experimental observation that the absolute rate of cyclisation is an important factor in determining the final conversion. As shown in Fig. 5, the initial rate of inhibitor formation is independent of the identity of the substrate, as would be expected if there is no significant influence of the aryl silane at the end of the tether on the rate of reaction of the trimethoxybenzene ring with the oxidant. The impact of this is that the cyclisation that proceeds with the fastest absolute rate will have the lowest percentage of inhibitor at a given time, and therefore will suffer least inhibition and attain greatest conversion before stalling (Fig. 7).

**Fig. 8:**
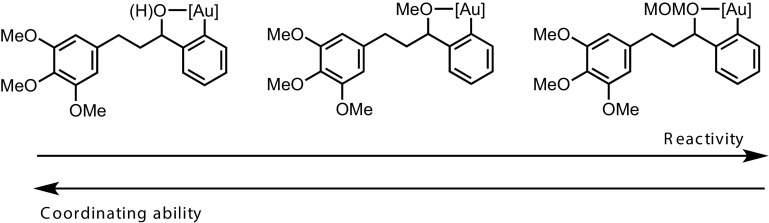
Possible origin of decreased cyclisation rate with more coordinating oxygen functuionality

The difference in turnover rate between MOM-protected **3**, methyl ether **18e**, and alcohol **18a**, can be tentatively attributed to the coordinating ability of the oxygen *ortho*- to the silane. If the oxygen can coordinate to the catalyst after the transmetalation (Fig. 8), this could serve to slow π-complexation of the arene to the gold, and thus the rate of cyclisation.

**Fig. 9:**
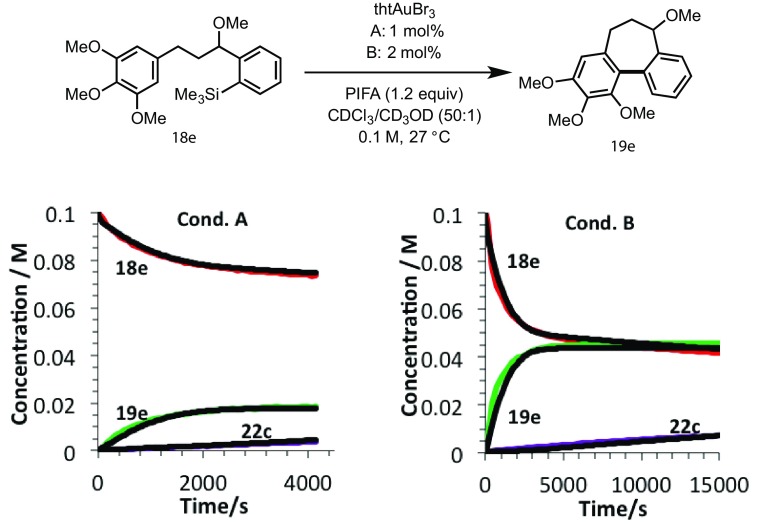
Kinetics of gold-catalysed cyclisation of **18e**. Conditions A: 1 mol%; Conditions B: 2 mol%. Black lines are simulated data based on the deactivation model shown in Scheme 10. The simulation correlates satisfactorily with experimental data for **18e, 19e** and **22c** when: *k*_*1*_:*k*_*2*_ = 1:5 (where *k*_*1*_, *k*_*2*_ > > *k*_*3*_), *k*_*3*_ = 0.014 s^−1^ (TLS) and *k*_*5*_ = 1.63 × 10^−4^ dm^3^ mol^−1^ s^−1^. For simplicity, *k*_*4*_ was set to an arbitrary value of > 1000 dm^3^ mol^−1^ s^−1^. See SI for full details

Using ^1^H NMR to monitor the formation of the proposed inhibitors (**22a**–**c**), kinetic modelling [18] can be used to deduce the partitioning between the productive cycle and the deactivation pathway (*k*_*1*_ / *k*_*2*_, Scheme 10). Using this model, a good fit for the deactivation of **18e** can be obtained for runs conducted with 1 and 2 mol% catalyst (Fig. 9)^4^. The model indicates that the in situ generated diaryliodonium salt (**22c**) is a powerful inhibitor, as *k*_*2*_ ≈ 5 × *k*_*1*_. It initially appears surprising that a distal diaryliodonium salt would have the effect of accelerating the rate of transmetalation at the silane. Our previous work suggests that the Au-catalyst intermediates are loosely-solvated, ionic species [2]. Accelerated transmetallation of the catalyst-inhibiting silane-tethered diaryliodonium salts may possibly involve salt metathesis, or localisation of a counter-anion for C-Si cleavage. Efforts to understand this process are on-going.

Although the oxidant change from HCIB (**1**) to PIFA (2) has allowed for the construction of new molecular structures by substantially attenuating diaryliodonium formation, the small amount (1–4%) of inhibitor that is still formed (Fig. 5) has been found to be significantly detrimental to the reaction (Fig. 9). We have also considered strategies to reduce the rate of the undesired diaryliodonium salt (**22**) generation by reducing the solution phase concentrations of the substrate or the oxidant, e.g. by slow addition techniques, or limiting rates of solid–liquid mass transfer. However, slow addition of oxidant or substrate both afforded poorer results, e.g. syringe-pump addition of a 0.1 M solution of **3** at a rate of 1 mol% per minute resulted in just 16% conversion to **4**, as compared to 70% conversion under the normal conditions (Fig. 6). This arises because of the onset of different catalyst deactivation pathways: the Au(I) to Au(III) redox that completes the catalytic cycle [2] requires both oxidant and arylsilane present to avoid Au-disproportionation, leading to catalytically inactive species.

It is thus clear from this study that moving away from hypervalent iodine oxidants to other species will be essential in expanding the generality and utility of the Au-catalysed arylation reaction [1–5]. Despite this, the methodology in its current state provides a number of advantages over other processes, e.g. those catalysed by Pd where high temperatures are often required [19, 20]. Moreover, the synthesis of allocolchicine (Scheme 1) clearly demonstrates how the orthogonality of the Au-catalysed arylation to Pd-catalysed cross-coupling [4] and functionalisation can aid in the synthesis of complex molecules.

**Scheme 11:**
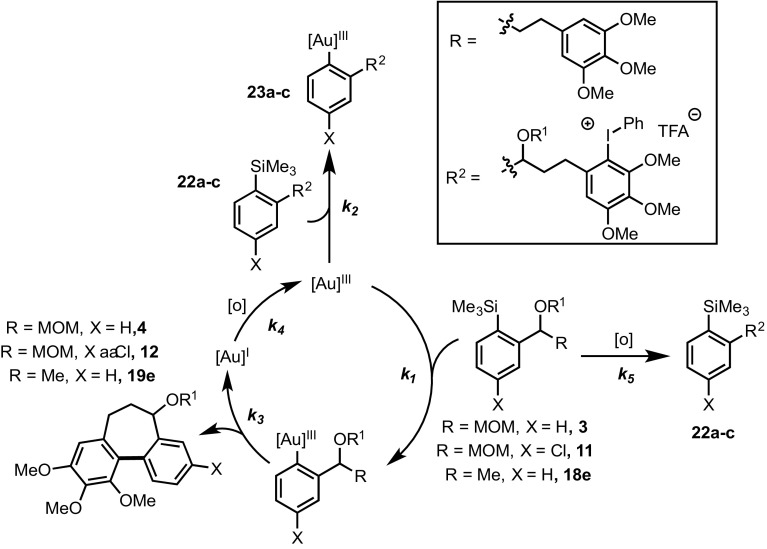
Proposed deactivation mechanism involving generation of tethered diaryliodonium salts **22a**–**c**

## Conclusion

In conclusion, we have developed a synthetic route to a number of allocolchinoid analogues, including a formal synthesis of (±)-allocolchicine (±)-**5** using a gold-catalysed direct arylation [1–6] in the key bond-forming step. The reaction is vulnerable to severe catalyst deactivation, with the likely cause identified as in situ inhibitor generation, involving a direct (uncatalysed) reaction of the substrate with the iodine(III) oxidant. The application of the process to complex molecule synthesis has led to new insights into Au(III) catalysis and has demonstrated both its strengths and weaknesses. We have shown that these complex highly electron rich molecules can be synthesised with operational simplicity and high turnover rates. However, the formation of side products can be seriously detrimental to the efficiency of the process by partial or complete progressive inhibition of the catalyst. The results reinforce the conclusion that finding alternatives to the use of hypervalent iodine oxidants will allow for significant improvements in the gold catalysed direct arylation methodology.

## Footnotes

1 CAS: 935-99-9.

2
**18c** and **18d** were assigned tentatively, **19d** was assigned *in situ* based on literature values (see ref. 7b)

3 See SI for full details.

4 Fits for the deactivation of **11** can be obtained with similar *k*_*1*_:*k*_*2*_ partitioning, a poor fit was obtained for the cyclisation of **3**.
